# Active billiards: Engineering boundaries for the spatial control of confined active particles

**DOI:** 10.1073/pnas.2426715122

**Published:** 2025-09-19

**Authors:** Roberto Di Leonardo, András Búzás, Lóránd Kelemen, Dávid Tóth, Szilvia Z. Tóth, Pál Ormos, Gaszton Vizsnyiczai

**Affiliations:** ^a^Dipartimento di Fisica, Sapienza Università di Roma, Rome I-00185, Italy; ^b^Institute of Nanotechnology, National Research Council of Italy, Soft and Living Matter Laboratory, Rome 00185, Italy; ^c^Institute of Biophysics, Biophotonics and Biomicrofluidics Research Group, Bionanoscience Research Unit, Hungarian Research Network Biological Research Centre, Szeged H-6726, Hungary; ^d^Institute of Plant Biology, Laboratory for Molecular Photobioenergetics, Plant Light Perception and Utilization Research Unit, Hungarian Research Network Biological Research Centre, Szeged H-6726, Hungary

**Keywords:** active matter, microswimmers, dynamical billiards

## Abstract

Unlike gas molecules at equilibrium, the spatial organization of self-propelled particles can be very sensitive to what happens at the boundaries of their container. Understanding the link between boundary phenomena and bulk stationary distributions could enable the design of optimized container shapes for the geometric control of confined active particles. Here, we propose a boundary method based on the flux transfer formalism typical of radiometry problems, where surface elements transmit and receive “rays” of active particles with infinite persistence length. We demonstrate the power of this boundary method in the case of the swimming microalgae *Euglena gracilis* trapped in light-defined billiard geometries. Quite surprisingly, we found that *Euglena* scatters with a nearly Lambertian cosine law, resembling the behavior of blackbody radiation and consequently resulting in nearly uniform distributions inside simple cavity geometries. Nevertheless, leveraging our boundary method, we were able to design a stacked multistage billiard geometry, with a connection scheme between subunits that breaks spatial symmetry and achieves an exponential amplification of cell concentration between its two ends. Our method can be applied to confined active matter in contexts ranging from spatial control and sorting of microorganisms to the design of efficient navigation strategies for microscopic and macroscopic robots.

## Significance

What photons, molecules, and microorganisms have in common is that, when confined within a cavity, they provide instances of a broad class of mathematical problems known as dynamical billiards. But if equilibrium imposes that photons and molecules should scatter off boundaries with Lambert’s “cosine law,” self-propelled particles, such as swimming microorganisms, defy this by exhibiting a wide variety of scattering behaviors, resulting in highly heterogeneous and boundary-sensitive spatial distributions. By adapting methods from radiometry, we link the shape of a boundary and the scattering behavior at its surface to the spatial distribution of particles inside. We applied this framework to guide swimming microalgae toward specific regions defined solely by the shape of the container.

An equilibrium gas will fill any container with a homogeneous density regardless of its shape and material. Conversely, nonequilibrium gases of self-propelled particles are extremely sensitive to boundary geometry and interactions. Asymmetric walls can produce currents and give rise to spontaneous accumulation (1–4), while the mechanical pressure exerted by an active gas over container walls can be strongly influenced by the details of interactions (5, 6). The connection between boundaries and bulk phases is extremely rich in active systems (7), including effects such as the appearance of Casimir-type forces (8), or the suppression of bulk phase separation by wall roughness (9). Moreover, if particle density is so sensitive to the boundary geometry, a feedback mechanism can emerge in which boundaries are actively remodeled by the density-dependent mechanical forces exerted by the particles (10, 11).

When active particles collide with a wall, the irreversible nature of self-propulsion results in scattering laws that break time reversal symmetry (12, 13). Microswimmers like *Escherichia coli* or catalytic Janus particles always end up to be aligned along the wall surface regardless of the incoming angle (14, 15). In contrast, ciliary contact interactions cause puller swimmers such as the single-celled algae *Chlamydomonas reinhardtii* to leave the surface at a constant angle, losing memory of incidence (16–18). This microscopic irreversibility has been exploited to design structured surfaces that could trap, sort, repel, and rectify the motion of microswimmers (1, 19–23). Moreover, thanks to a rich sensory apparatus, the concept of boundary for living systems is broader than just physical walls. Chemotactic bacteria are pushed out of regions where repellents are present, while photosensitive microorganisms can bounce back at abrupt transitions between light and dark (24).

Given the crucial role of boundaries in determining bulk properties, and the wide variety of particle-wall scattering behaviors, there is a clear need for new modeling frameworks that can provide a quantitative connection between the spatial distribution of active particles, the geometry of the confining boundary, and their specific scattering dynamics at the walls. On the practical side this could guide the engineering of boundary shapes to control the internal spatial distribution of active particles, transport and segregate cell types based on wall interactions, or simply inhibit unwanted accumulation for biofilm prevention. A theoretical framework for the steady states of active Brownian particles near boundaries was proposed in ref. 25. This analytical approach relies on the diffusion approximation, which restricts its validity to regimes where the persistence length is much smaller than the characteristic length scale of the boundary geometry. Since the persistence length of many microswimmers can reach several tens of microns, this framework offers only a good approximation when applied to macroscopic confinement geometries. Moreover, for complex boundary geometries, analytical solutions are generally not accessible, and numerical approaches require solving partial differential equations on a finite element discretization of the entire domain enclosed by the boundaries. In the opposite regime, where the confinement size is small compared to the persistence length, an analytical relation between particle distribution and boundary shape can be derived for the simple case in which walls do not affect the direction of particle self-propulsion (26, 27). In this limit, all particles accumulate on the boundary in the steady state, with a density proportional to the local curvature, while the bulk remains empty. However, to reproduce experimental observations one must incorporate boundary scattering and previous studies have typically addressed this through particle-based simulations (18, 28). Thus, despite the progress made, a general framework that can predict the steady-state distribution of real active particles, taking into account their specific scattering laws and arbitrary confinement geometries, is still lacking.

Here, we focus on a class of active particles that move along straight trajectories and undergo memoryless scattering processes at the boundary of an arbitrarily shaped region. Using a formalism borrowed from radiometry, we show that the problem of finding the steady state spatial distribution of these particles can be cast into a boundary problem to be solved numerically. To verify the correctness of our model, we study how different scattering laws produce different density modulations depending on the shape of the boundary and compare these theoretical results with numerical simulations of active particles. In many equilibrium situations, like photons in a blackbody cavity or rarefied molecules in a Knudsen gas (29), scattering follows a Lambertian cosine law, which in the case of active particles also results in perfectly uniform distributions inside cavities of any shape. Deviations from Lambert’s law result in regions of high concentration that shift from the bulk to the boundary as the scattering angles change from normal to tangential directions. In addition, we provide a direct experimental application of our method to a system of flagellated microalgae *Euglena gracilis* swimming in optically defined arenas. Upon encountering a light–dark interface, light-responsive Euglena cells undergo random scattering events that reorient the cells toward the cavity interior and keep them confined. We found that Euglena scatters from light–dark interfaces with a nearly Lambertian law so that cells spread almost uniformly inside simple cavity shapes. Nevertheless, using the results of our boundary method, we were able to design a stacked multistage structure that results in a three-fold concentration of Euglena cells between its two ends. Model predictions are quantitatively confirmed by experiments, demonstrating the accuracy of our method and its practical applications.

## 1. A Boundary Element Method for Confined Active Particles

**Fig. 1.**
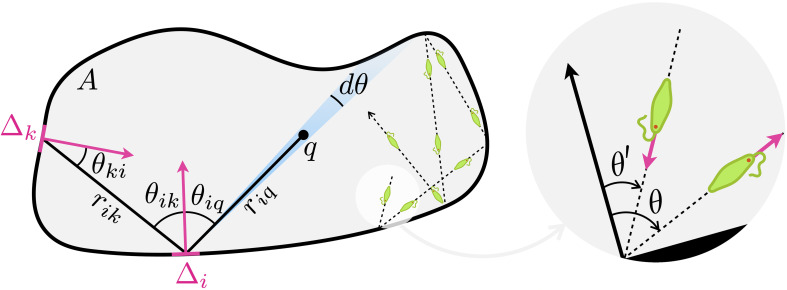
Geometric description of the boundary model.

When the persistence length of active particles exceeds the size of the container, they will travel in straight lines from one element of the boundary to the next. Every time a particle “feels” a boundary through mechanical, hydrodynamical, chemical, or optical signals it quickly reorients to a new swimming direction. We call $S(\theta^{\prime },\theta )$ the scattering law representing the probability density of being scattered to a new direction θ given the incident angle $\theta^{\prime }$. We will assume that the boundary is impenetrable so that both θ and $\theta^{\prime }$, defined as in Fig. 1, vary between $-90^{{}^{\circ}}$ and $90^{{}^{\circ}}$.

Then we discretize the boundary in finite elements of lengths $\Delta_{i}$ and call $J_{i}\left(\theta \right)d\theta$ the stationary flux of particles emerging from the i-th boundary element within an angle dθ centered around θ. This flux results from scattered particles arriving in $\Delta_{i}$ from all other elements $\Delta_{k}$ so that we can write:

[1] $$\begin{array}{c} J_{i}\left(\theta \right)\Delta_{i}=\sum_{k\neq i}J_{k}\left(\theta_{\mathrm{ki}}\right)\Delta_{k}\frac{\Delta_{i}\cos\theta_{\mathrm{ik}}}{r_{\mathrm{ik}}}S\left(\theta_{\mathrm{ik}},\theta \right). \end{array}$$

Although, in principle, the above equation could be solved for $J_{i}\left(\theta \right)$ for a given scattering law $S(\theta^{\prime },\theta )$ (for instance by discretizing $J_{i}\left(\theta \right)$ into an array of values for equally spaced angles between $-90^{{}^{\circ}}$ and $90^{{}^{\circ}}$ and so turning it into a linear algebra problem), the solution simplify noticeably when active particles lose memory during the scattering event so that $S\left(\theta^{\prime },\theta \right)=\sigma \left(\theta \right)$. Substituting in Eq. **1**, we get

[2] $$\begin{array}{c} J_{i}\left(\theta \right)\Delta_{i}=\sigma \left(\theta \right)\sum_{k\neq i}J_{k}\left(\theta_{\mathrm{ki}}\right)\Delta_{k}\Delta_{i}\frac{\cos\theta_{\mathrm{ik}}}{r_{\mathrm{ik}}}=\sigma \left(\theta \right)\Delta_{i}p_{i} \end{array}$$

with

[3] $$\begin{array}{c} p_{i}\Delta_{i}=\sum_{k\neq i}J_{k}\left(\theta_{\mathrm{ki}}\right)\Delta_{k}\Delta_{i}\frac{\cos\theta_{\mathrm{ik}}}{r_{\mathrm{ik}}}=\sum_{k\neq i}W_{\mathrm{ik}}p_{k}\Delta_{k}, \end{array}$$

where we introduced the variables $p_{i}$ representing the stationary flux of particles colliding/emerging on boundary element i per unit time and unit length. The matrix $W_{\mathrm{ik}}=\sigma \left(\theta_{\mathrm{ki}}\right)\Delta_{i}\cos\theta_{\mathrm{ik}}/r_{\mathrm{ik}}$ represents the probability that a particle scattered from boundary element k will hit next at i so that it must satisfy the normalization rules $\sum_{i}W_{\mathrm{ik}}=1$. The matrix $W_{\mathrm{ik}}$ is fully determined by the geometry of the boundary and the scattering law. In concave boundaries, the connection of segment pairs can be occluded, for which case $W_{\mathrm{ik}}$ must be set to 0. Once these are fixed the $p_{i}$ values can be found by solving the linear algebra problem in Eq. **3** that in matrix form reads:

[4] $$\begin{array}{c} \left(\begin{array}{cccc} -\Delta_{1} & W_{12}\;\Delta_{2} & \cdots & W_{1n}\;\Delta_{n}\\ W_{21}\;\Delta_{1} & -\Delta_{2} & \cdots & W_{2n}\;\Delta_{n}\\ \vdots & \vdots & \ddots & \vdots \\ W_{n1}\;\Delta_{1} & W_{n2}\;\Delta_{2} & \cdots & -\Delta_{n} \end{array}\right)\cdot \left(\begin{array}{c} p_{1}\\ p_{2}\\ \vdots \\ p_{n} \end{array}\right)=\left(\begin{array}{c} 0\\ 0\\ \vdots \\ 0 \end{array}\right). \end{array}$$

Once the boundary fluxes $p_{i}$ have been determined the particle density at a generic internal point q with coordinates r=(x,y) can be obtained as the sum of density contributions from all elements:

[5] $$\begin{array}{c} \rho \left(r\right)=\sum_{i=1}^{n}p_{i}\Delta_{i}\frac{\sigma (\theta_{\mathrm{iq}})}{vr_{\mathrm{iq}}}, \end{array}$$

where v is the particle speed. A possible way of seeing it is shown in Fig. 1 where $p_{i}\Delta_{i}\sigma \left(\theta_{\mathrm{iq}}\right)d\theta$ represents the flow of particle emerging from element $\Delta_{i}$ within an angle dθ around the direction $\theta_{\mathrm{iq}}$. This flow can be also written as $\rho_{i}\left(r\right)v\;r_{\mathrm{iq}}\;d\theta$, where $\rho_{i}\left(r\right)$ is the density of particle emitted from $\Delta_{i}$. Form that we find $\rho_{i}\left(r\right)=p_{i}\Delta_{i}\sigma \left(\theta_{\mathrm{iq}}\right)/vr_{\mathrm{iq}}$ and summing over all elements we get the total density in Eq. **5**. The values $p_{i}$ derived from Eq. **4** are defined within an arbitrary multiplicative factor so that we can absorb the factor v in the $p_{i}$ values and normalize in the end by imposing the condition $\int_{A}\rho \left(r\right)d^{2}r=1$, where A is the surface enclosed by the boundary.

## 2. The Role of Geometry and Scattering Law

As a first application of our boundary method, we investigate how the combined effects of boundary shape and scattering function affect the spatial distribution of active particles in the interior. We choose four different geometries: a generic shape, a circle, an ellipse, and an equilateral triangle. For every geometry, we consider the six different scattering laws in Table 1. The first one represents isotropic scattering, particles emerge with an angle that is uniformly distributed between −90 and 90 degrees. The second, third, and fourth scattering laws describe cases where particles emerge with an increasingly narrow distribution around the surface normal, represented mathematically as the cosine of the outgoing angle raised to increasingly larger powers. The fifth case describes particles that are preferentially aligned parallel to the wall while the last one corresponds to a shallow scattering angle as found for *Chlamydomonas* algae scattering from solid surfaces.

**Table 1.** List of the examined scattering laws

1	1	isotropic	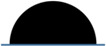
2	cosθ	Lambert	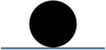
3	$\cos^{2}\theta$	normal	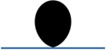
4	$\cos^{8}\theta$	normal narrow	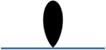
5	1−cosθ	wall aligning	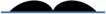
6	${\left(1-\cos3\theta \right)}^{4}$	shallow angle	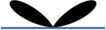

**Fig. 2.**
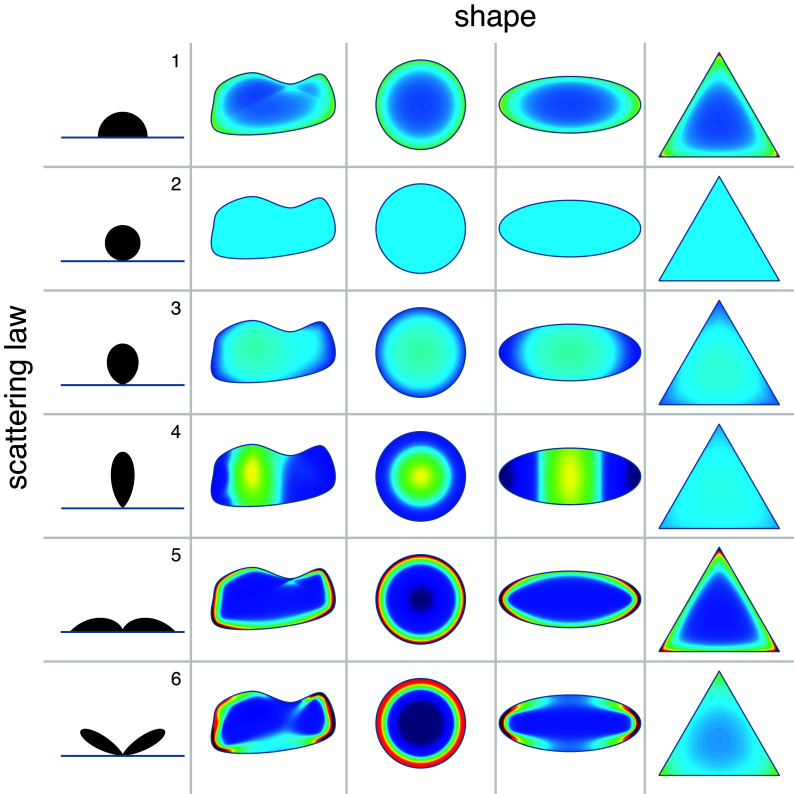
The role of geometry and scattering law. Spatial probability density distributions calculated with the boundary method in four different confinement geometries for the set of scattering angle distributions defined in Table 1.

Solving the boundary problem, we obtain spatial distributions of active particles within the cavity that are very sensitive to both geometry and scattering law (Fig. 2). As expected, the wall-aligning and the shallow angle scattering law result in a high probability of finding active particles close to the boundary, favoring regions of higher convex curvature. Results for shallow angle scattering in the circle and ellipse confinements also match with the previously reported experimental observations of single *Chlamydomonas* microalga cells in such compartments (28, 30). Wall accumulation is a general feature of active particles and is often the consequence of slowing down due to wall repulsion counteracting self-propulsion (31). But here we are considering instantaneous scattering events with particles that do not slow down when they hit the boundary, so it is somewhat surprising to find this wall accumulation even for isotropic scattering. In contrast, scattering distributions that favor outgoing directions close to the surface normal tend to generate concentration peaks in the inner region, while maintaining a low density at the edge. Interestingly, in the equilateral triangle very narrow and normal directed scattering results in an almost uniform distribution. Another notable exception is when the scattering probability is in the form of cosθ (number 2 in Table 1), which produces a uniform concentration for any geometry. Interestingly, this kind of particle scattering is analogous to Lambert’s cosine law for photons, where the cosine shaped scattering makes the brightness of Lambertian surfaces invariant to the observation angle. It also characterizes the emission of radiation from the surface of a blackbody cavity, a consequence of the isotropic radiation (uniform energy density) inside the cavity. In terms of our boundary model, when the scattering law is σ(θ)=cosθ/2 then we have the detailed balance condition:

[6] $$\begin{array}{c} W_{\mathrm{ik}}\Delta_{k}=\frac{\cos\theta_{\mathrm{ki}}\cos\theta_{\mathrm{ik}}\Delta_{i}\Delta_{k}}{2r_{\mathrm{ik}}}=W_{\mathrm{ki}}\Delta_{i} \end{array}$$

so that Eq. **3** admits the constant solution $p_{i}=p^{*}$

[7] $$\begin{array}{c} p^{*}\Delta_{i}=\sum_{k\neq i}W_{\mathrm{ik}}p^{*}\Delta_{k}=\sum_{k\neq i}W_{\mathrm{ki}}p^{*}\Delta_{i}=p^{*}\Delta_{i}\sum_{k\neq i}W_{\mathrm{ki}}, \end{array}$$

where the identity follows from the normalization condition $\sum_{k\neq i}W_{\mathrm{ki}}=1$. A uniform boundary flux $p^{*}$ produces a uniform bulk density ρ(r) as can be easily deduced from Eq. **5** noting that for Lambert scattering $\Delta_{i}\cos\theta_{\mathrm{iq}}/r_{\mathrm{iq}}$ is just the angular size of element $\Delta_{i}$ from point q.

**Fig. 3.**
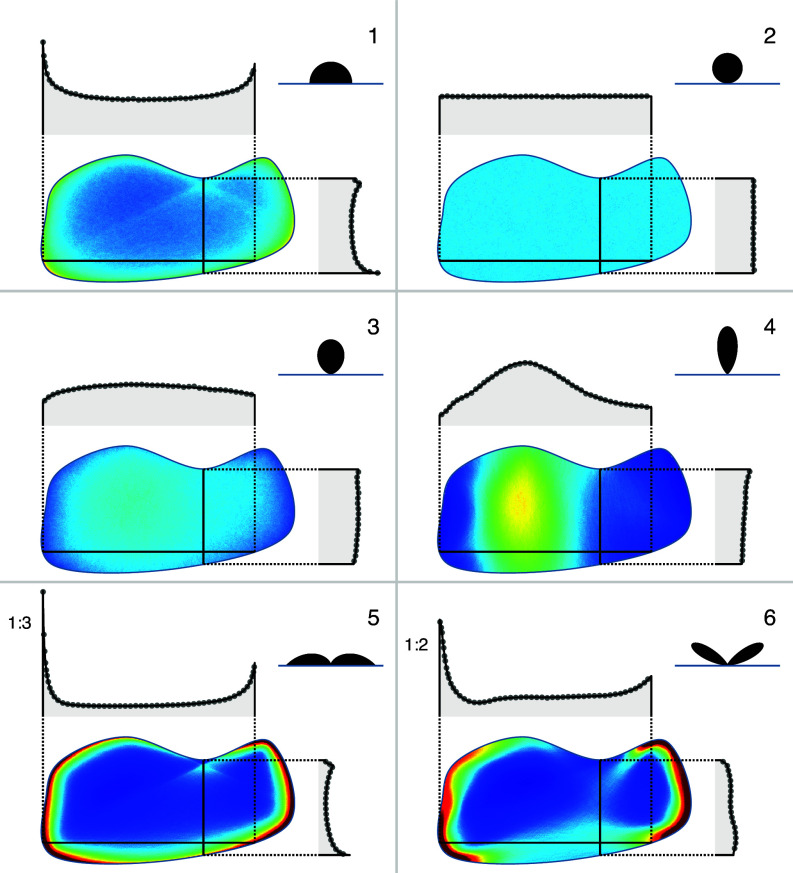
Validation of the boundary method with active particle simulations. Two-dimensional spatial probability density distributions obtained with particle simulations are shown for a generic shape and a set of scattering laws. Results of the two methods are compared on plots of density profiles along two sample lines, with the simulation results represented by gray circles and the boundary model results by black lines.

To validate the results of our method we have performed numerical simulations of active particle dynamics (*Materials and Methods*) whose results are reported for a selected geometry in Fig. 3. As shown by density profiles along two sample lines the agreement between theory and simulation is perfect.

## 3. Distributions of Euglena in Optical Confinement

We now want to test the predictive power of our method in a real system composed of the flagellated photosynthetic alga *E. gracilis*. *Euglena* are unicellular microorganisms with an elongated body shape of approximately 50 μm in length and 10 μm diameter. As their single flagellum beats at 20 to 40 Hz, they swim at 50 to 140 μm s^−1^, while simultaneously rolling at 1 to 2 Hz along their longitudinal axis (32).

**Fig. 4.**
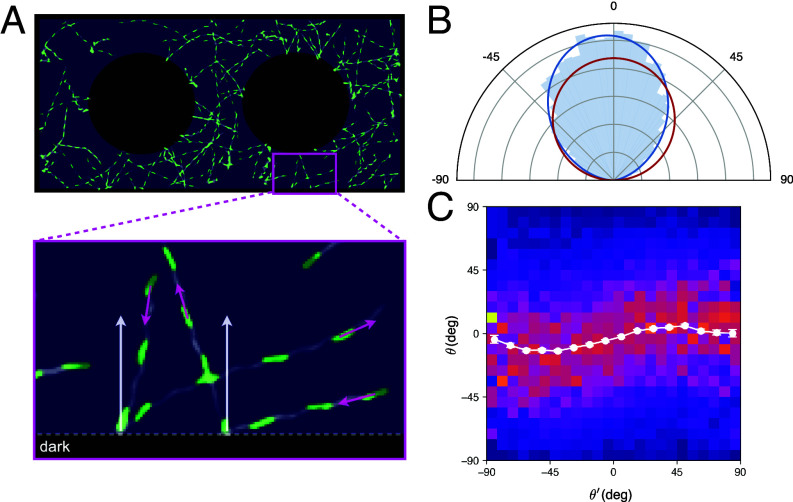
Scattering angle distribution of *E. gracilis* cells. (*A*) Image of the measurement’s light pattern with a sample timelapse of swimming cells superimposed. The enlarged area shows a zoomed view on two cells going through scattering at the light–dark boundary. White arrows depict the local boundary normal. (*B*) Polar histogram of the measured scattering angles. The blue line plots the best fit with $\cos^{\alpha }\left(\theta +\theta_{0}\right)$, while the red line shows the Lambert cosine law. (*C*) Dependence of the outgoing angle θ from the incoming angle $\theta^{\prime }$. The white plot shows the mean outgoing angle in respective intervals of the incoming angle.

*Euglena* cells are known to be trapped in regions of light surrounded by darkness if the illumination intensity is not too high (33). The flagellar beat can change in response to light variations detected by a photoreceptor located at the base of the flagellum (34) resulting in an inverse photophobic response at the boundary of the illuminated region, where the cells are essentially scattered back inward. Using a blue light projection system with a properly adjusted intensity, we confined cells into two-dimensional light domains with reconfigurable geometries (Movie S1). Within a light pattern, the cells swim in straight paths until they reach the boundary between light and dark. Here, they perform a stochastic rotation until they find a new swimming direction pointing inward. We have recorded a large number (∼20,000) of these scattering events within a rectangular light patch with two circular holes included to increase the probability of observing scattering events with shallow angles of incidence (Fig. 4*A*) (Movie S2). We track individual cells and analyze trajectories to fully characterize the scattering law $S(\theta^{\prime },\theta )$ reported in Fig. 4*C* as a density map. The outgoing angle distributions, represented by the columns in the density map, displays a weak dependence on the incoming angle $\theta^{\prime }$. Therefore, memoryless scattering, $S\left(\theta^{\prime },\theta \right)=\sigma \left(\theta \right)$, seems to be a good approximation for *Euglena*. In that approximation σ(θ) can be directly obtained as the outgoing angle distribution averaged over all incoming angles. The result is shown as a polar histogram in Fig. 4*B* together with a Lambert cosine law (red line) and a best fit representation with the model $\cos^{\alpha }\left(\theta +\theta_{0}\right)$ (blue line, best fit parameters α=1.7 and $\theta_{0}=4.2$). This scattering law is roughly intermediate between Lambert cosine and normal scattering (models 2 and 3 in Table 1). Based on the previous discussion, we would expect to find a mild accumulation in the interior of the light region.

**Fig. 5.**
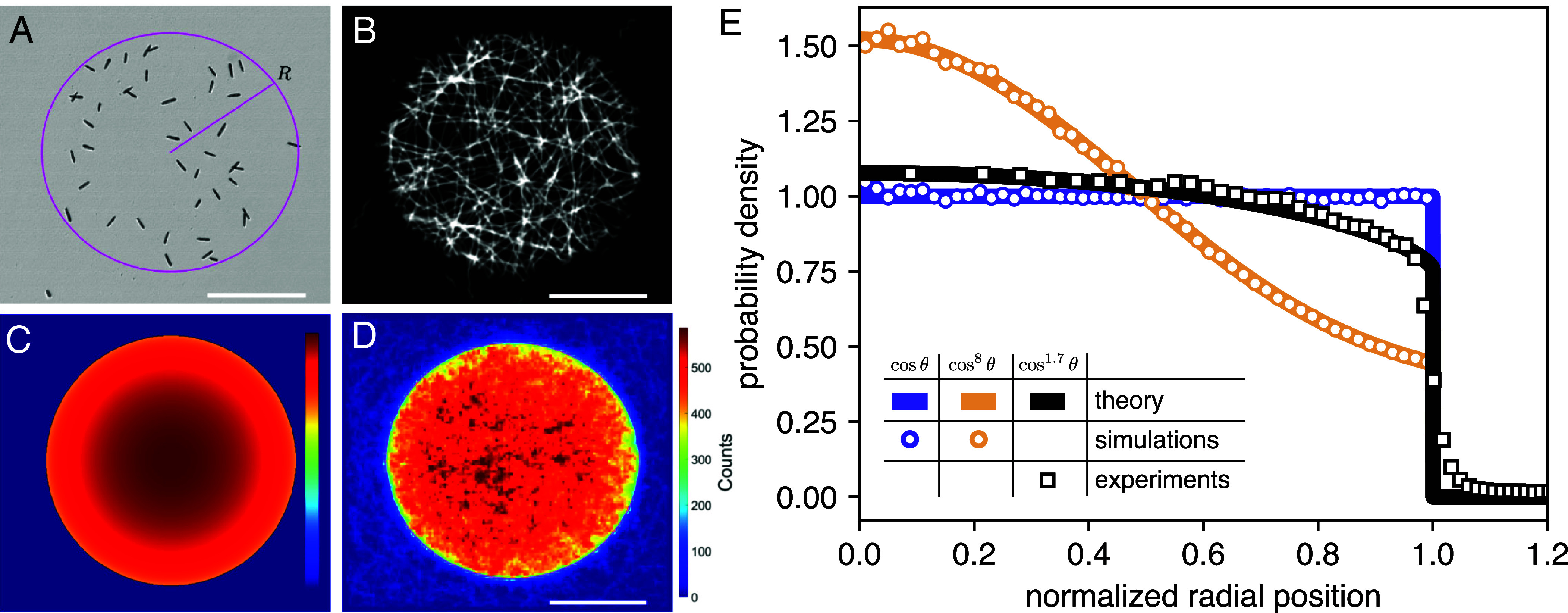
Spatial distribution of *E. gracilis* cells confined in a circular light patch. (*A*) Bright-field image snapshot of cells during a measurement. The light pattern’s edge is marked by the magenta line. (*B*) A random sample of persistent cell trajectories. (*C*) Boundary model results. (*D*) Two-dimensional spatial distribution of the cells (smoothed with a 0.5 bin wide Gaussian). (*E*) Radial spatial probability density distributions calculated from measurement data (black squares) and from simulation results of particles with scattering distributions of cosθ (purple circles) and $\cos^{8}\theta$ (orange circles). Theoretically calculated density curves are shown as solid lines. (Scale bars, 0.5 mm.).

As a first test of our boundary method we studied *Euglena* distribution inside a circular region of radius R=630μm (Fig. 5*A* and Movie S3). A sample of the measured cell trajectories is shown in Fig. 5*B*, obtained after stuck and strongly curving trajectories were removed by a trajectory filter (*Materials and Methods* and *SI Appendix*, Fig. S1). At a first qualitative sight, the experimental 2D density map (Fig. 5*D*) shows a slight depletion of cells at the boundary which compares well with the boundary method prediction shown in (Fig. 5*C*). For a more quantitative comparison, we make a radial histogram of cell density for which we can also derive a semianalytical prediction from our model. Assuming isotropic dynamics, symmetry considerations imply a uniform boundary flux p and a stationary density that only depends on the distance r from the center. Rewriting Eq. **5** in integral form, we get

[8] $$\begin{array}{c} \rho \left(r\right)=p\int \frac{\sigma (\theta )}{d}ds=2p\int_{0}^{\pi }\frac{\sigma (\theta )}{\cos\theta }d\phi , \end{array}$$

where the variable θ can be expressed as a function of ϕ and r using the condition Rsinθ=rsinϕ. For scattering laws of the form $\sigma \left(\theta \right)\propto \;\cos^{m}\theta$ we get

[9] $$\begin{array}{c} \rho \left(r\right)=Z\int_{0}^{\pi }{\left[1-{\left(\frac{r}{R}\right)}^{2}\sin^{2}\phi \right]}^{\frac{m-1}{2}}d\phi \end{array}$$

with Z a normalization factor. By numerical integration, we obtain the density profiles for n=1,8 which agree perfectly with simulation results as shown in Fig. 5*E*. On the same figure we also show experimental data for *Euglena* together with the corresponding theoretical prediction for m=1.7. The agreement is remarkably good in all interior points. The experimental density profile however displays a softer decay to zero at r=R which we attributed to the cell’s light sensing apparatus. As in many unicellular algae, the photoreceptor is periodically shaded by a light absorber (eyespot) as the cell rolls during forward swimming. This produces a finite resolution in sensing the location of dark to light transition which we can estimate as the distance traveled in a half rotation during which the cell is “blind.” Using average values for speed (98 μm /s) and rolling frequency (0.8 Hz), we get a distance of 62 μm, or about 0.1 R, which is enough to account for the extent of the smooth transition at the circle boundary.

## 4. Amplification of Cell Concentration in Pattern Sequences with Broken Spatial Symmetry

**Fig. 6.**
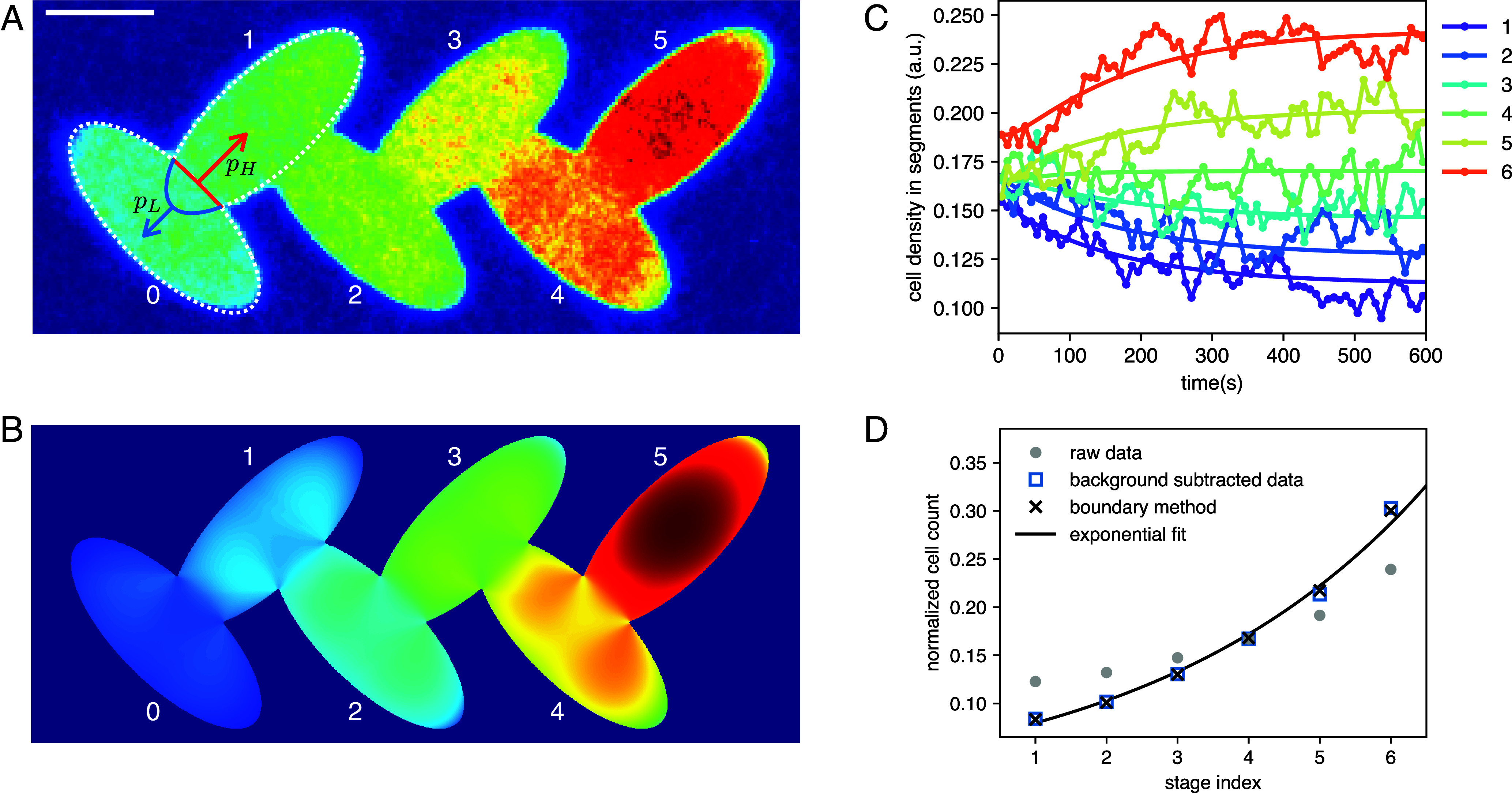
Rectification of *E. gracilis* cells confined in a two-dimensional light pattern. (*A*) Measured steady state density map of *Euglena* cells (smoothed with a 0.5 bin wide Gaussian). (Scale bar, 0.5 mm.) (*B*) Boundary model result. (*C*) Time evolution of the normalized cell counts within the light pattern’s segments (lines with markers) and their fit with Eq. **12** (solid lines). (*D*) Normalized cell counts in the pattern’s segments in steady-state (t > 250 s).

Due to the scattering law for *Euglena* differing only slightly from the Lambert cosine case, we observed a rather flat cell concentration profile in circular confinement. The average density within a central disk of radius R/2 is only 13% higher than in the outer region. Looking at other shapes in Fig. 2, we find similar mild concentration enhancements in the central region for scattering laws close to *E*uglena (row no. 3). However, if we focus on the ellipse, we find a noticeable depletion region at the high curvature poles (left and right) compared to the low curvature poles (top and bottom). From Eq. **5**, we see that when we move close to a generic boundary segment i, local density will be dominated by the total flux $p_{i}$ through that i segment. This consideration suggests a possible strategy to reach higher concentrations of cells by joining together equal shapes. If we start with a shape that, like the ellipse, shows an evident p variation along the boundary, we can connect a high p edge in one shape to a low p edge in a following shape, and so on. For ellipses, this means connecting them as in Fig. 6 by repeatedly overlapping low and high curvature poles and thereby creating a sequential pattern that breaks spatial symmetry.

We have performed experiments with two horizontally mirrored versions of the designed sequential ellipse pattern, collecting tens of millions of cell position data points. Testing the pattern in two orientations and merging the results allowed us to exclude any additional effects that might bias the cell’s movement. Measurements were started with approximately uniform cell densities in each ellipse by turning on the light pattern projection over areas of homogeneously spread cells. Starting from uniformity, the cell count in each ellipse stage evolves over time until the system reaches steady state (Fig. 6*C*). Let us assume that the net flow of cells across the connections of neighboring ellipses is small enough that their spatial distribution remains close to equilibrium as the integrated cell number in each area slowly varies. Calling $N\left(t\right)=\left\{N_{0},\cdots ,N_{5}\right\}$ the vector of normalized cell counts in each area at time t, we can write

[10] $$\begin{array}{c} \dot{N}\left(t\right)=K\cdot N\left(t\right) \end{array}$$

with K the tridiagonal matrix

[11] $$\begin{array}{c} K_{i,i}=-\left(\lambda_{+}+\lambda_{-}\right)\;,\;\;K_{i,i+1}=\lambda_{-}\;,\;\;K_{i,i-1}=\lambda_{+} \end{array}$$

with $\lambda_{+}$ and $\lambda_{-}$ are respectively forward and backward transition rates and with boundary conditions $K_{11}=-\lambda_{+}$, $K_{66}=-\lambda_{-}$. The formal solution is

[12] $$\begin{array}{c} N\left(t\right)=e^{Kt}\cdot N\left(0\right), \end{array}$$

which can be used to fit the time series of measured normalized cell counts in Fig. 6*C*.

In the stationary state the net flux over each junction must be zero

[13] $$\begin{array}{c} N_{n}\lambda_{+}-N_{n+1}\lambda_{-}=0 \end{array}$$

resulting in a cell count growing exponentially after each stage

[14] $$\begin{array}{c} N_{n}=N_{0}{\left(\frac{\lambda_{+}}{\lambda_{-}}\right)}^{n}. \end{array}$$

The ratio $\lambda_{+}/\lambda_{-}$ can be estimated by our previous investigation on an isolated ellipse. Suppose we have two initially disjoint and equilibrated ellipses each one containing N cells. The moment we connect them as ellipses number 0 and 1 in Fig. 6*A* a larger number of cells will cross from 0 to 1 in unit time ($p_{H}$) than in the opposite direction ($p_{L}$). The values of $p_{H}$ and $p_{L}$ can be estimated as the cumulative values of $p_{i}\Delta_{i}$ over the overlapping sections of the low and high curvature boundaries of the isolated ellipses (Fig. 6*A*), respectively. Since $p_{H}≃N\lambda_{+}$ and $p_{L}≃N\lambda_{-}$, we obtain that $\lambda_{+}/\lambda_{-}≃p_{H}/p_{L}=1.45$ for the *Euglena* scattering law. The measured steady state (t > 250 s) density map is shown in Fig. 6*A*. Similar to the analysis of circle trajectories, a filter was used to remove trajectory portions corresponding to stationary cells because they were stuck or turning at the edge. The spatial distribution of cells matches very well the prediction of the boundary model (Fig. 6*B*): within each ellipse, cell concentration is depleted at the high curvature poles while it progressively increases as we move from left to right. It is worth noting that the left–right symmetry is broken by the specific connection scheme, in which flat poles are joined to curved poles as the segment index i increases. For a quantitative comparison we count the number of cells in each elliptical segment and plot it as gray circles in Fig. 6*D*. The boundary model (black crosses) seems to predict a larger concentration gradient than what was found experimentally. However, if we subtract from raw data a modest (half of the smallest count) background value and renormalize (blue squares), we get an almost perfect agreement with boundary model predictions. The uniform density background may result from a small fraction of cells with low persistence lengths (*SI Appendix*, Fig. S2) and new cells entering the pattern from outside, without having had sufficient time to be influenced by the shape of the confinement. Normalized cell counts can be very well fitted by the exponential law [**14**] with a fitted value for $\lambda_{+}/\lambda_{-}=1.3$ which is satisfactorily close to 1.45 that was theoretically estimated above from the ratio $p_{H}/p_{L}$.

## 5. Conclusions

The nonequilibrium nature of active systems allows for new spatial control strategies that are not available in equilibrium systems. While boundary shape is generally irrelevant for the bulk distribution of confined equilibrium systems, we demonstrate that active particles can be spatially organized solely by acting on the boundary geometry. Here, we provide a boundary method that combines the geometric shape of the container with the particle-specific scattering law at the container walls to make fast and accurate predictions of how active particles distribute in a confined environment. We applied this method to an active system composed of *E. gracilis* microalgae cells, which bounce like active billiards off the walls of regions whose geometry can be dynamically and arbitrarily reconfigured with light. We demonstrated that the proposed boundary method enables fast and accurate prediction of experimental cell distributions. Building on these results, we designed a static periodic structure that breaks spatial symmetry in a way that is specifically tailored for the directed transport of active particles with a scattering law like that of *Euglena*. As a result, we observed an exponential amplification of cell concentration, leading to a threefold increase in a six-stage structure, as predicted by our boundary method.

Our results expand our understanding of the propagation and distribution of active particles in confined spaces, enabling control over their arrangement through the shape of the container. From a fundamental point of view, this expands the field of dynamical billiards to include active systems for which the scattering laws are stochastic and not constrained by the laws of equilibrium physics (35). Unlike classical deterministic billiards, the presence of stochasticity guarantees ergodicity so that densities are always well defined. As a result, this class of billiards offers a valuable framework for understanding, and controlling, how complex biological systems explore space.

Future research directions include the design of boundary geometries that efficiently sort active particles based on their distinct scattering behavior, or that generate targeted flow patterns in confined spaces. To this end, our method could serve as the basis for a general framework for solving the inverse problem of determining the optimal geometry that maximizes certain figures of merit, such as the density ratio between two sites. In this context, it will be important to address fundamental questions such as what constraints a given scattering law imposes on the creation of arbitrarily complex patterns. Finally, the proposed boundary method approach may facilitate the engineering of custom scattering laws for programmable micro- and macroscopic robots, enabling controlled navigation and exploration of complex or unknown spaces (36).

## 6. Materials and Methods

### 6.1. Numerical Implementation of the Boundary Method.

We have implemented our boundary method in MATLAB and CUDA. We have written two CUDA GPU kernels for the calculation of the matrix W and for the calculation of the in-confinement spatial probability density distributions using Eq. **5**. The CUDA kernels were directly called from MATLAB using the ptx programming model. To use the CUDA GPU’s best performance, we have chosen to use single precision in the GPU code. A standard desktop computer was used with an NVIDIA TITAN XP GPU.

### 6.2. Active Particle Simulation.

In the simulation, a point-like particle is moved in finite steps inside a confining polygon, such that when the particle reaches the boundary, it will be scattered into a new swimming direction defined by an angle relative to the boundary normal, that is chosen randomly from a given angular probability distribution. The final result of the simulation is the spatial position distribution of the particle calculated from the accumulated position data.

### 6.3. E. gracilis Cultures.

Cultures were grown mixotrophically in Tris-acetate-phosphate medium (37) (TAP) in 25-mL Erlenmeyer flasks on a rotatory shaker at 130 rpm, at 23 ^°^C and 80 μmol photon m^−2^ s^−1^. The cultures were transferred to fresh TAP medium every 2 wk, and 1-wk-old cultures were used for the experiments.

### 6.4. E. gracilis Experiments.

We have performed experiments with *E. gracilis* in a custom built, but simple optical setup. We used a Texas Instruments DLP^®^ LightCrafter™ DM365 digital light projector with a blue LED (470 nm) light source to project binary intensity patterns onto the sample of the cells over an 8-by-4 mm area. Illumination intensity was 0.4 µW /${\mathrm{mm}}^{2}$ to achieve positive phototactic response from the cells. Cells were placed in samples consisting of two microscope coverglasses separated by 100 μm thick double sided tape. Imaging was performed under red light illumination with a Point Grey Grasshopper3 USB3 (GS3-U3-23S6M-C) camera at a magnification of 1×.

The persistence length of the cells was measured by tracking cells trapped in a rectangular light pattern of 4.2 × 1.5 mm size. The persistence length of the measured trajectories was obtained by fitting a Worm-like chain model:

[15] $$\begin{array}{c} \langle R^{2}\rangle =2PL\left[1-\frac{P}{L}\left(1-e^{-L/P}\right)\right], \end{array}$$

where $\langle R^{2}\rangle$ and L are the mean squared end-to-end distance and the length of a trajectory, and P is the persistence length. Trajectories with lengths shorter than 0.5 mm were not considered in the analysis. Results are shown on *SI Appendix*, Fig. S2.

### 6.5. Cell Tracking and Trajectory Filtering.

Cells were tracked with a custom software written in MATLAB, that is capable of tracking cells even when they swim over each other. The recorded bright-field images were background subtracted and inverted to obtain dark background images with bright cells. After a threshold operation, image objects are matched to already existing trajectories. Unmatched objects are assigned to new trajectories, unless the object’s morphological parameters (area, solidity) do not match that of a single cell. Image objects that got matched to multiple trajectories are further processed as overlapping cell objects. These overlapping cells are segmented with a contour curvature segmentation algorithm designed to detect the cell’s ends. From the detected cell-ends, new cell positions are calculated and are then matched to the prematched trajectories considering both the position and the orientation of the detections and the trajectories.

To remove stuck and low persistence cells from our trajectory data, we applied a trajectory filter. Here, a trajectory is first segmented by its heading angle using MATLAB’s ischange function. This way we are able to segment a trajectory into moving and turning segments. Then, for each segment, we calculate the slope of the trajectory’s direction angle, which we use to remove turning cells undergoing scattering and cells whose trajectory direction changes rapidly. To remove stuck cells we also apply a speed filter, where the mean speed of with which a trajectory segment is going away from its starting point is calculated. For the circle measurement, we used a trajectory angle slope threshold of 15 degree/s, while for the ratchet measurements, we used a value of 60 degree/s. The speed-filter threshold was 40 μm/s for both types of measurements.

## Supplementary Material

Appendix 01 (PDF)

Movie S1. Confining *Euglena gracilis* cells into a light pattern. The video shows *Euglena gracilis* collecting into a binary checkerboard light pattern, recorded with dark field imaging, shown at 2x speed.

Movie S2. Scattering angle measurement of *Euglena gracilis* cells. The video shows *Euglena gracilis* cells swimming confined inside a light pattern composed of a bright rectangle with 2 dark circles inside. Background subtracted and inverted bright-field images of the cells are blended with the fluorescence image (blue) of the illumination pattern. Shown at 8x speed.

Movie S3. *Euglena gracilis* cells swimming confined into a circular light pattern. Background subtracted and inverted bright-field images of the cells are blended with the fluorescence image (blue) of the illumination pattern. Shown at 8x speed.

Movie S4. *Euglena gracilis* cells swimming confined into a sequential ellipse light pattern. Background subtracted and inverted bright-field images of the cells are blended with the fluorescence image (blue) of the illumination pattern. Shown at 8x speed.

## Data Availability

All original data consist of large size video recordings that we will make available to anyone upon request.
