# Supplementary material for: Active billiards: Engineering boundaries for the spatial control of confined active particles
Source: Proc Natl Acad Sci U S A. 2025 Sep 19;122(38):e2426715122. doi: 10.1073/pnas.2426715122 (PMC12478192; doi:10.1073/pnas.2426715122)
Supplement: Supplementary file 1 — Appendix 01 (PDF) [file pnas.2426715122.sapp.pdf]

PNAS

Supporting Information for

Active billiards: connecting boundary shape and dynamics to spatial distributions

Roberto Di Leonardo, András Búzás, Lóránd Kelemen, Dávid Tóth, Szilvia Z. Tóth, Pál Ormos, Gaszton Vizsnyiczai

Corresponding Author: Roberto Di Leonardo and Gaszton Vizsnyiczai.
E-mail: roberto.dileonardo@uniroma1.it and vizsnyiczai.gaszton@brc.hu

This PDF file includes:

Figs. S1 to S2
Legends for Movies S1 to S4

Other supporting materials for this manuscript include the following:

Movies S1 to S4

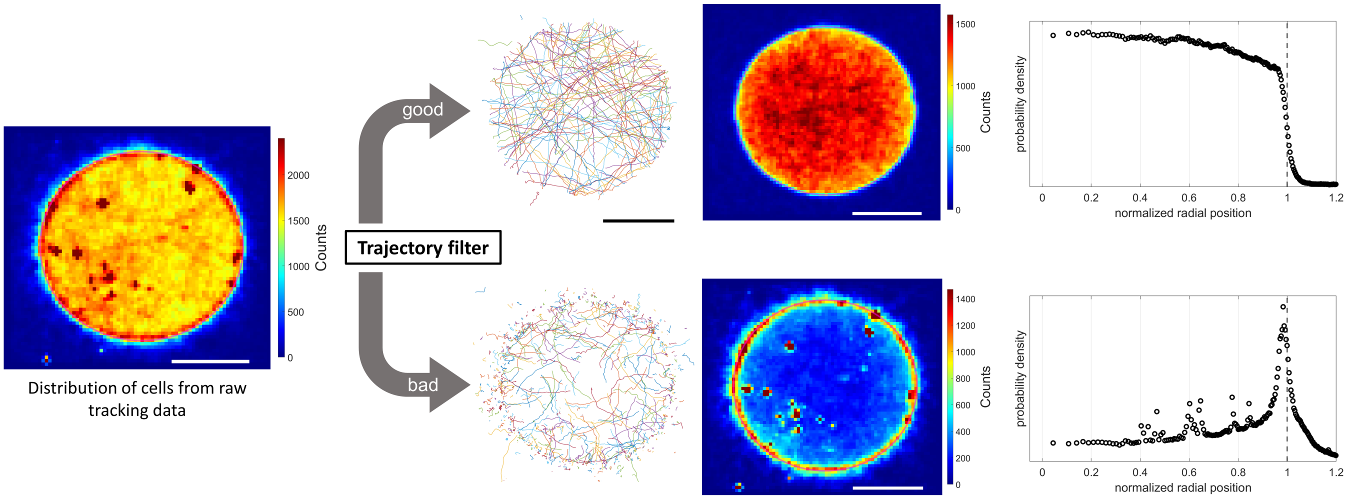

Fig. S1. Demonstration of trajectory filtering. Position distribution of cells from raw tracking data is polluted by stuck cells and a background from low persistence length trajectories. A trajectory filter extracts straight trajectories from the measurements. A sample of trajectory plots, the corresponding 2D and radial cell distributions are shown for the accepted and rejected trajectory data respectively. Scalebars 0.5 mm.

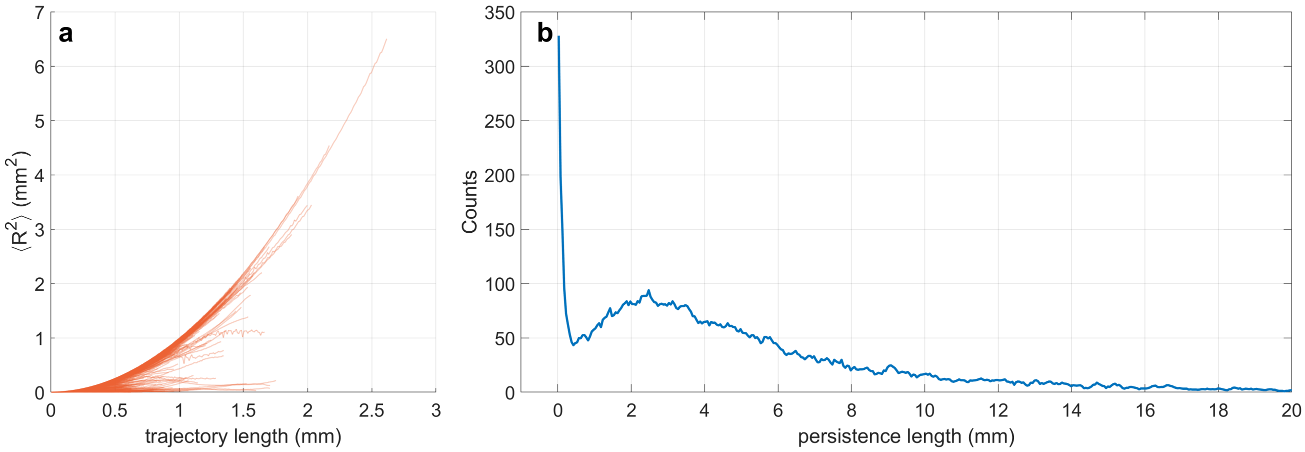

Fig. S2. Persistence lengths of swimming *Euglena gracilis* cells. **a** Plots of the mean squared end-to-end distance in respect to the total length for each measured trajectory. **b** Distribution of the fitted persistence lengths. About 8% of the cells show a very low persistence length, shown by the peak in the histogram in the [0, 0.5] mm interval.

Movie S1. Confining *Euglena gracilis* cells into a light pattern. The video shows *Euglena gracilis* collecting into a binary checkerboard light pattern, recorded with dark field imaging, shown at 2x speed.

Movie S2. Scattering angle measurement of *Euglena gracilis* cells. The video shows *Euglena gracilis* cells swimming confined inside a light pattern composed of a bright rectangle with 2 dark circles inside. Background subtracted and inverted bright-field images of the cells are blended with the fluorescence image (blue) of the illumination pattern. Shown at 8x speed.

Movie S3. *Euglena gracilis* cells swimming confined into a circular light pattern. Background subtracted and inverted bright-field images of the cells are blended with the fluorescence image (blue) of the illumination pattern. Shown at 8x speed.

Movie S4. *Euglena gracilis* cells swimming confined into a sequential ellipse light pattern. Background subtracted and inverted bright-field images of the cells are blended with the fluorescence image (blue) of the illumination pattern. Shown at 8x speed.
